# Delay-Sensitive NOMA-HARQ for Short Packet Communications

**DOI:** 10.3390/e23070880

**Published:** 2021-07-09

**Authors:** Faisal Nadeem, Mahyar Shirvanimoghaddam, Yonghui Li, Branka Vucetic

**Affiliations:** Centre for IoT and Telecommunications, School of Electrical and Information Engineering, The University of Sydney, Sydney, NSW 2006, Australia; mahyar.shm@sydney.edu.au (M.S.); yonghui.li@sydney.edu.au (Y.L.); branka.vucetic@sydney.edu.au (B.V.)

**Keywords:** finite blocklength, HARQ, non-orthogonal multiple access, ultra-reliable and low latency communication

## Abstract

This paper investigates the two-user uplink non-orthogonal multiple access (NOMA) paired with the hybrid automatic repeat request (HARQ) in the finite blocklength regime, where the target latency of each user is the priority. To limit the packet delivery delay and avoid packet queuing of the users, we propose a novel NOMA-HARQ approach where the retransmission of each packet is served non-orthogonally with the new packet in the same time slot. We use a Markov model (MM) to analyze the dynamics of the uplink NOMA-HARQ with one retransmission and characterize the packet error rate (PER), throughput, and latency performance of each user. We also present numerical optimizations to find the optimal power ratios of each user. Numerical results show that the proposed scheme significantly outperforms the standard NOMA-HARQ in terms of packet delivery delay at the target PER.

## 1. Introduction

Most of the advancements in wireless cellular communication, such as third-generation (3G), fourth-generation (4G) and 4G long-term evolution (LTE), are primarily focused on the human-centered communication for enabling enhanced mobile broadband (eMBB) communication [1]. The fifth-generation (5G) of mobile standards envisions to include massive machine-type communications (mMTC) and ultra-reliable low-latency communications (URLLC) into its area of focus, apart from eMBB [2,3]. In eMBB, usually, the file size is large, and the packet reliability is given priority over the packet level latency [4]. URLLC would be a key enabler of various mission-critical applications, such as telesurgery, tactile Internet, factory automation, and smart grids [5]. URLLC traffic is delay-sensitive; therefore, a delayed packet is considered as an erroneous packet. URLLC has two conflicting performance requirements, i.e., low latency, which requires user plane latency below 1 ms, and high reliability, which requires a packet error rate (PER) less than $10^{-6}$ for a packet of size 32 bytes, with or without retransmission [5,6]. mMTC is the key enabler of the Internet of Things applications, such as smart metering, smart agriculture, etc. These services involve massive connectivity and low-power communications to support billions of devices, mainly transmitting short messages [7]. Energy efficiency and massive connectivity are required to enable mMTC. Therefore, mMTC design should be scalable and supportive of providing various latency and reliability performance [8] to an immense number of devices, which would increase to 50 billion by 2030 [9].

In 5G, short packet communications are considered to be an effective way to enable low-latency communications for URLLC and mMTC applications. Conventional communication protocols are mainly designed based on the Shannon’s capacity formula, which is suitable only when the packet length is considered infinite. Such designs usually lead to significant performance losses when the packet length is short [7]. Recently, several performance bounds have been developed for error rate performance in the finite blocklength regime, e.g., the normal approximation [10]. In particular, the coding gain is reduced at finite blocklengths, as packets experience a finite number of channel observations and the gap to Shannon’s limit is increased [6,10]. To compensate for the coding gain, available diversity sources, such as space and frequency, can be utilized [11,12]. Due to limited resources and the fact that many URLLC applications will operate over unlicensed bands, frequency diversity is not a viable solution. Instead, one can utilize retransmission techniques, such as hybrid automatic repeat request (HARQ) [13,14], which increase latency [15,16].

Non-orthogonal multiple access (NOMA) schemes can be used to exploit channel diversity and resource utilization by simultaneously allocating a channel to multiple users [17]. NOMA has been actively investigated in the past decade, since it can effectively provide higher throughput and flexibility in comparison to orthogonal multiple access (OMA) techniques [7,18,19]. In NOMA, multiple devices can share the same radio resources using the superposition of signals. Successive interference cancellation (SIC) is used to separate the signal of each user at the receiver [20]. NOMA can be implemented by either power or code sharing between users [21]. NOMA is mainly analyzed in the asymptotic blocklength regime and, more recently, in the finite blocklength regime, proving that NOMA effectively provides better resource utilization and energy efficiency [22,23,24]. In [22,25], the authors show that NOMA outperforms OMA and reduces the latency under a finite blocklength regime. In NOMA, more users can be served over limited channel resources, resulting in higher spectral efficiency, which reduces latency as well [22]. These features of NOMA make it a potential candidate technique for URLLC and mMTC scenarios. In HARQ, in case of packet failure, the receiver feedbacks a negative acknowledgment (NACK) and requests for retransmission. In contrast, upon receiving an ACK, the transmitter sends a new packet. Upon a retransmission request, the transmitter can either send a duplicate copy of the packet, known as chase combining HARQ (CC-HARQ), or send more redundancy through forward error correcting code, known as incremental redundancy HARQ (IR-HARQ) [26]. The receiver combines the retransmission with failing packets to increase the decoding reliability. With CC-HARQ, maximum ratio combing (MRC) is used to increase the effective signal-to-noise ratio (SNR), whereas with IR-HARQ, code combing is used to increase reliability.

In the asymptotic blocklength regime, when the channel is perfectly known at the transmitter, rate adaptation via adaptive modulation and coding can be used to reduce the retransmission requests [27]. However, in the finite blocklength regime, the retransmission requests are more probable due to the high error rate of finite length codes [28]. Both IR-HARQ and CC-HARQ are actively being investigated in the finite blocklength regime [13,15]. The delay performance is optimized for single user with HARQ in the finite blocklength regime in the Rayleigh fading channel in [29]. HARQ was recently analyzed with NOMA in the downlink set up with two users in [30,31,32,33], where the outage performance was analyzed in the infinite blocklength regime with rate and power adaptation. HARQ-enabled NOMA is also studied in [22,23] to evaluate its usefulness in enabling URLLC and mMTC. In [34], the authors analyzed HARQ in an uplink NOMA setting, focusing on enabling retransmissions to be distinguishable from the regular transmission to facilitate grant-free HARQ communication. Moreover, in [35], the authors adjust power levels among users to reduce retransmission requests. Retransmission with HARQ causes additional delays in communication. Efforts have been made to improve retransmission quality resulting in throughput gain [36]. However, the throughput gain only translates to average delay performance improvements [37]. URLLC and many mMTC scenarios require low-latency with a per-packet delay guarantee.

In this paper, we consider an uplink NOMA system paired with HARQ for short packet communications, where the target per-packet latency of each user is the priority. Although the HARQ process improves reliability, it increases latency and causes packet queuing. The primary motivation of this work is to increase reliability without causing latency by maintaining per-packet arrival deadlines. We propose a novel NOMA-HARQ approach, where the retransmission of each packet is served non-orthogonally with the new packet in the same time slot. We use a Markov model (MM) to analyze the dynamic of the uplink NOMA-HARQ with one retransmission and characterize the PER, throughput, and latency performance of each user. We also present numerical optimizations to minimize PER and find the optimal power ratios of each user. Numerical results show that the proposed scheme significantly outperforms the standard NOMA-HARQ in terms of the packet delivery latency at the target PER.

The rest of the paper is organized as follows. In Section 2, the system model and preliminaries on NOMA and HARQ are presented. Section 3 presents the proposed delay-sensitive NOMA-HARQ scheme, where its reliability and delay analysis are discussed. Section 4 presents numerical results. Finally, Section 5 concludes the paper.

## 2. System Model and Preliminaries

We consider an uplink power-domain NOMA scenario, where $N_{u}$ users can simultaneously send their messages to the base station (BS). Similar to [38], we consider time division duplex system (TDD) so that BS synchronizes the uplink transmission of each user by sending a beacon signal at the beginning of each time slot. The channel between the *i*-th user, $1\leq i\leq N_{u}$, and the BS, denoted by $g_{i}$, is modeled by large-scale path-loss and small-scale Rayleigh fading [39]. We assume that BS knows the channel state information (CSI) of each user perfectly. The *i*-th user encodes and modulates its $k_{i}$-bit message into a packet of length *n* symbols and sends it to the BS. Let y(t) denote the received signal at the BS at time *t* given as:

(1) $$y\left(t\right)=\sum_{i=1}^{N_{u}}g_{i}x_{i}\left(t\right)+w\left(t\right),$$

where $x_{i}\left(t\right)\in C$ is the transmitted complex symbols from the *i*-th user and w(t)∼CN(0,1) is the additive white Gaussian noise (AWGN). We assume that $E[|x_{i}\left(t\right){|}^{2}]=1$. Let $P_{i}$ be the received power of user *i* at the BS given as

(2) $$\begin{array}{c} P_{i}={\left|g_{i}\right|}^{2}P_{t,i}, \end{array}$$

where $P_{t,i}$ is the transmit power of user *i*, $|g_{i}{|}^{2}=h_{i}r_{i}^{-\rho },$$h_{i}$ is the small-scale fading with exponential distribution, i.e., $h_{i}∼\exp\left(1\right)$, $r_{i}$ is the distance between the *i*-th user and the BS, and ρ is the path-loss exponent. We assume block fading channel model, such that the channel remains constant over a time block and changes independently between the blocks.

**Figure 1:**
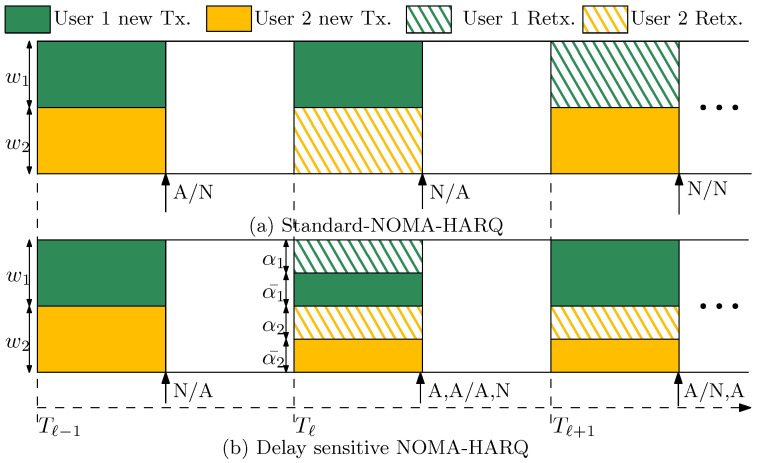
Packetization model of two-user NOMA-HARQ with single retransmission.

The BS can pair users according to their CSIs and SNR levels to meet the desired level of reliability. BS usually pairs near and far users to exploit their power difference for better SIC decoding. We assume that paired users have certain finite channel gains, so that their transmit power does not exceed the maximum energy budget. Let $0<w_{i}<1$ denote the ratio of powers between the paired users, such that $P_{i}=w_{i}P_{c}$, where $P_{c}$ denotes the total received power at the BS from paired users, where $w_{1}+w_{2}=1$ (We use parameter $w_{i}$ to simplify presentation regarding effect of power difference in the total received power. Otherwise, if the total transmit power constraint on each user is used we need to specify channel gains while presenting the results.), when $N_{u}=2$. We assume that $w_{1}>w_{2}$ to treat user 1 as a near user and user 2 as a far user. In practical settings, there may be channel estimation errors that could lead to degradation in optimal user pairing as well as the SIC performance of multiple access systems (In future publications, we would incorporate channel estimation errors and its impact on the performance.). The receiver first decodes user 1 while treating the message of other users as noise. If user 1’s signal is successfully recovered, it is then removed from the received signal and user 2’s signal is then decoded and removed from the received signal. This continues to decode all $N_{u}$ users. Each user reports its decoding status using an instantaneous ACK. Upon receiving an ACK, the user sends a new packet; otherwise, upon receiving a NACK, it retransmits the previous packet, through either CC-HARQ or IR-HARQ, in the next time slot. Generally, user 1 is decoded first due to its higher received power at the BS, unless other users have more copies due to retransmissions. Figure 1a shows packet transmission with NOMA and standard HARQ (S-NOMA-HARQ) [22] when $N_{u}=2$.

We use normal approximation [10], to characterize the PER in the finite blocklength regime. For CC-HARQ, the bound in [10] can be used with accumulated SNR after MRC, as follows:

(3) $$\begin{array}{c} \epsilon_{\mathrm{cc}}\left(\Gamma^{\left(m\right)}\right)\approx Q\left(\frac{n\log_{2}\left(1+\sum_{j=1}^{m}\gamma_{j}\right)-k_{i}+\log_{2}\left(n\right)}{\sqrt{nV(\sum_{i=j}^{m}\gamma_{j})}}\right), \end{array}$$

and the bound in [40] for parallel AWGN channels can be used to calculate the PER for IR-HARQ [40], as follows:

(4) $$\begin{array}{c} \epsilon_{\mathrm{ir}}\left(\Gamma^{\left(m\right)}\right)\approx Q\left(\frac{n\sum_{j=1}^{m}\log_{2}\left(1+\gamma_{j}\right)-k_{i}+\log_{2}\left(mn\right)}{\sqrt{n\sum_{i=j}^{m}V\left(\gamma_{j}\right)}}\right), \end{array}$$

where $\Gamma^{\left(m\right)}=\left[\gamma_{1},\cdots ,\gamma_{m}\right]$ is the vector of signal to interference plus noise ratios (SINRs) for *m* copies of a packet, $V\left(\gamma_{j}\right)=\left(1-{\left(1+\gamma_{j}\right)}^{-2}\right)\log_{2}^{2}\left(e\right)$ is the channel dispersion and Q(.) is the standard *Q*-function. $k_{i}$ is the length of user *i*’s message and *n* is the length of the codeword in each transmission. Accordingly, the rate of user *i* in the first transmission is $R_{i}=k_{i}/n$.

## 3. Delay-Sensitive NOMA-HARQ

As can be seen in Figure 1a, in S-NOMA-HARQ, each *i*-th user conducts its retransmission with its maximum power in the new time slot. This causes new arriving packets to be delayed when retransmission is requested. We propose a delay-sensitive NOMA-HARQ (D-NOMA-HARQ) designed for delay-sensitive applications that avoid the excess delay due to retransmissions and adhere to the packet deadlines. More specifically, user *i* conducts its retransmission with its new arriving packet non-orthogonally. That is, when retransmission is requested from user *i*, it will superimpose the retransmission packet and new packet with power fractions $\alpha_{i}$ and $\bar{\alpha_{i}}$, respectively, where $\bar{\alpha_{i}}=1-\alpha_{i}$. The D-NOMA-HARQ scheme for two users with a maximum one retransmission, is shown in Figure 1b.

### 3.1. Reliability and Throughput Analysis of Two-User D-NOMA-HARQ

**Figure 2:**
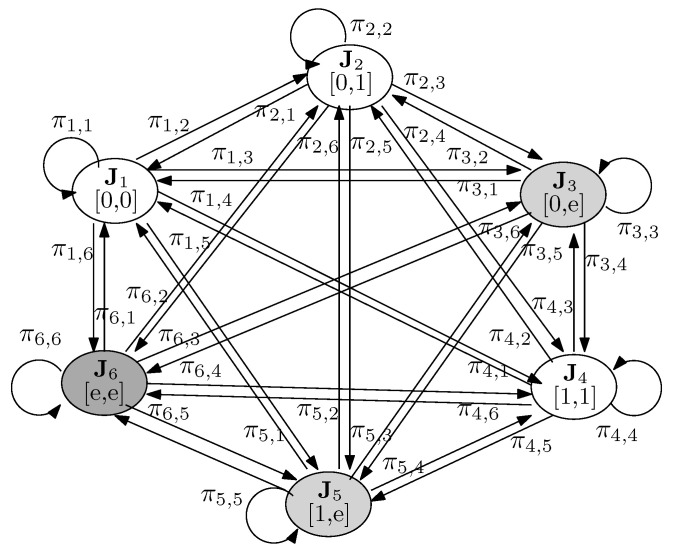
Markov model of the proposed D-NOMA-HARQ with two users and a single retransmission.

We use an MM, as shown in Figure 2, whose states are represented by a vector $J=[J_{1},J_{2}]$, where $J_{i}\in \left\{0,1,e\right\}$ is the current state of user *i*. State 0 refers to a packet success without any retransmission, State 1 refers to a packet success after single retransmission, and State *e* refers to packet failure after single retransmission.

It is important to note that as each user can be in one of the possible three states, the MM for the proposed D-NOMA-HARQ when $N_{u}=2$ with single retransmission will have 9 states. However, since we assume that user 1 is a near user and it will always transmit with a higher power and is decoded first at the receiver, and if unsuccessful user 2 will not be decoded, the number of possible states will reduce to 6. In particular, when $J_{1}=0$, $J_{2}$ can be 0,1, or *e*, which corresponds to vector states $J_{1}$, $J_{2}$, and $J_{3}$, respectively. When $J_{1}=1$, $J_{2}$ can only be 1 or *e*, which corresponds to states $J_{4}$ and $J_{5}$. This is because of the assumption that user 1 is always transmitting with a higher power, i.e., $w_{1}>w_{2}$ and $\alpha_{1}>\alpha_{2}$, where $\alpha_{i}$ is the fraction of retransmission power. This assumption simplifies the SIC decoding order without compromising performance. Otherwise, if each user freely chose $w_{i}$ and $\alpha_{i}$ in the range [0,1], the BS needs to find the optimal decoding order based on the received powers in each time slot with additional complexity [22]. Therefore, under this assumption, if user 1 is successful only after single retransmission, user 2 cannot be recovered with single transmission. Following a similar argument, when $J_{1}=e$, $J_{2}$ can be only *e*, which corresponds to state $J_{6}$ in the MM in Figure 2. The state transition probabilities of the MM are given in the following lemma.

**Lemma** **1.** *For the two-user D-NOMA-HARQ with a maximum of 1 retransmission, i.e., m=2, the probability of transitioning from state $J_{u}$ to state $J_{v}$, denoted by $\pi_{u\to v}$, ∀u,v∈{1,2,3,4,5,6} is given by*

$$\pi_{u\to v}=\left\{\begin{array}{cc} \left(1-\epsilon \left(\gamma_{u}^{\left(1\right)}\right)\right)\left(1-\epsilon \left(\gamma_{u}^{\left(2\right)}\right)\right), & v=1\\ \left(1-\epsilon \left(\gamma_{u}^{\left(1\right)}\right)\right)\left(\epsilon \left(\gamma_{u}^{\left(2\right)}\right)-\epsilon \left(\left[\gamma_{u}^{\left(2\right)},{\tilde{\gamma }}_{1}^{\left(2\right)}\right]\right)\right), & v=2\\ \left(1-\epsilon \left(\gamma_{u}^{\left(1\right)}\right)\right)\epsilon \left(\left[\gamma_{u}^{\left(2\right)},{\tilde{\gamma }}_{1}^{\left(2\right)}\right]\right), & v=3\\ \left(\epsilon \left(\gamma_{u}^{\left(1\right)}\right)-\epsilon \left(\left[\gamma_{u}^{\left(1\right)},{\tilde{\gamma }}_{1}^{\left(1\right)}\right]\right)\right)\left(1-\epsilon \left(\left[\gamma_{u}^{\left(2\right)},{\tilde{\gamma }}_{2}^{\left(2\right)}\right]\right)\right), & v=4\\ \left(\epsilon \left(\gamma_{u}^{\left(1\right)}\right)-\epsilon \left(\left[\gamma_{u}^{\left(1\right)},{\tilde{\gamma }}_{1}^{\left(1\right)}\right]\right)\right)\epsilon \left(\left[\gamma_{u}^{\left(2\right)},{\tilde{\gamma }}_{2}^{\left(2\right)}\right]\right), & v=5\\ \epsilon \left(\left[\gamma_{u}^{\left(1\right)},{\tilde{\gamma }}_{1}^{\left(1\right)}\right]\right), & v=6 \end{array}\right.$$

*where $\gamma_{u}^{\left(i\right)}$ denotes the SINR corresponding to the i-th user at the u-th vector state, ${\tilde{\gamma }}_{z}^{\left(i\right)}$ denotes the SINR of i-th user during retransmission, where z∈{1,2} indicates variation in SINR due to different combination of users states. The SINRs are given by ${\tilde{\gamma }}_{1}^{\left(1\right)}=\frac{\alpha_{1}P_{1}}{P_{2}+{\bar{\alpha }}_{1}P_{1}+1}$, ${\tilde{\gamma }}_{1}^{\left(2\right)}=\frac{\alpha_{2}P_{2}}{P_{1}+{\bar{\alpha }}_{2}P_{2}+1}$, ${\tilde{\gamma }}_{2}^{\left(2\right)}=\frac{\alpha_{2}P_{2}}{{\bar{\alpha }}_{1}P_{1}+{\bar{\alpha }}_{2}P_{2}+1}$, $\gamma_{1}^{\left(1\right)}=\frac{P_{1}}{P_{2}+1}$, $\gamma_{1}^{\left(2\right)}=P_{2}$, $\gamma_{2}^{\left(1\right)}=\frac{P_{1}}{{\bar{\alpha }}_{2}P_{2}+1}$, $\gamma_{2}^{\left(2\right)}={\bar{\alpha }}_{2}P_{2}$, $\gamma_{3}^{\left(1\right)}=\frac{P_{1}}{P_{2}+1}$, $\gamma_{3}^{\left(2\right)}=\frac{{\bar{\alpha }}_{2}P_{2}}{\alpha_{2}P_{2}+1}$, $\gamma_{4}^{\left(1\right)}=\frac{{\bar{\alpha }}_{1}P_{1}}{{\bar{\alpha }}_{2}P_{2}+1}$, $\gamma_{4}^{\left(2\right)}=\gamma_{2}^{2}$, $\gamma_{5}^{\left(1\right)}=\frac{{\bar{\alpha }}_{1}P_{1}}{P_{2}+1}$, $\gamma_{5}^{\left(2\right)}=\frac{{\bar{\alpha }}_{2}P_{2}}{\alpha_{2}P_{2}+1}$, $\gamma_{6}^{\left(1\right)}=\frac{{\bar{\alpha }}_{1}P_{1}}{P_{2}+\alpha_{1}P_{1}+1}$, and $\gamma_{6}^{\left(2\right)}=\frac{{\bar{\alpha }}_{2}P_{2}}{\alpha_{1}P_{1}+\alpha_{2}P_{2}+1}$. Moreover, ϵ(.) is given in (3) and (4) for CC-HARQ and IR-HARQ respectively.*

**Proof**  Similar to ([41], Lemma 1), we know that $1-\epsilon \left(\gamma_{u}^{\left(i\right)}\right)$, $\epsilon \left(\gamma_{u}^{\left(i\right)}\right)-\epsilon \left(\gamma_{u}^{\left(i\right)},{\tilde{\gamma }}_{1}^{\left(1\right)}\right)$, and $\epsilon \left(\gamma_{u}^{\left(i\right)},{\tilde{\gamma }}_{1}^{\left(1\right)}\right)$ are representing the probabilities that packet of user *i* is decoded without retransmission, with one retransmission, or is failed the decoding, respectively. These correspond to the probabilities of user *i* being at states $J_{i}=0$, $J_{i}=1$, and $J_{i}=e$, respectively. Secondly, the state transition probability for states $J_{u}$ (u=[1,⋯,6]) is the product of marginal probabilities of each user’s state $J_{i}$. For example, when $J_{1}=0$ and $J_{2}=0,1$ or *e*, the system state transits from $J_{u}$ to $J_{1}$, $J_{2}$ and $J_{3}$ with probabilities $\left(1-\epsilon \left(\gamma_{u}^{\left(1\right)}\right)\right)\left(1-\epsilon \left(\gamma_{u}^{\left(2\right)}\right)\right)$, $\left(1-\epsilon \left(\gamma_{u}^{\left(1\right)}\right)\right)\left(\epsilon \left(\gamma_{u}^{\left(2\right)}\right)-\epsilon \left(\left[\gamma_{u}^{\left(2\right)},{\tilde{\gamma }}_{1}^{\left(2\right)}\right]\right)\right)$ and $\left(1-\epsilon \left(\gamma_{u}^{\left(1\right)}\right)\right)\epsilon \left(\left[\gamma_{u}^{\left(2\right)},{\tilde{\gamma }}_{1}^{\left(2\right)}\right]\right)$ respectively. When user 1 is successful after a single retransmission, i.e., $J_{1}=1$ then user 2 can only be in two states i.e., $J_{2}=1$ or *e*. Consequently, the marginal probabilities of $J_{2}=1$ and $J_{2}=e$ when $J_{1}=1$ are $\left(1-\epsilon \left(\left[\gamma_{u}^{\left(2\right)},{\tilde{\gamma }}_{2}^{\left(2\right)}\right]\right)\right)$ and $\epsilon \left(\left[\gamma_{u}^{\left(2\right)},{\tilde{\gamma }}_{2}^{\left(2\right)}\right]\right)$, respectively. Therefore, the system state transits from $J_{u}$ to $J_{4}$ and $J_{5}$ with probabilities $\left(\epsilon \left(\gamma_{u}^{\left(1\right)}\right)-\epsilon \left(\left[\gamma_{u}^{\left(1\right)},{\tilde{\gamma }}_{1}^{\left(1\right)}\right]\right)\right)\left(1-\epsilon \left(\left[\gamma_{u}^{\left(2\right)},{\tilde{\gamma }}_{2}^{\left(2\right)}\right]\right)\right)$ and $\left(\epsilon \left(\gamma_{u}^{\left(1\right)}\right)-\epsilon \left(\left[\gamma_{u}^{\left(1\right)},{\tilde{\gamma }}_{1}^{\left(1\right)}\right]\right)\right)\epsilon \left(\left[\gamma_{u}^{\left(2\right)},{\tilde{\gamma }}_{2}^{\left(2\right)}\right]\right)$, respectively. Similarly, the system state transits form $J_{u}$ to $J_{e}$ with probability $\epsilon \left(\left[\gamma_{u}^{\left(1\right)},{\tilde{\gamma }}_{1}^{\left(1\right)}\right]\right)$. This is due to the fact that when user 1 is in error, user 2 is for sure in error.

Note that, in D-NOMA-HARQ, the *i*-th user conducts its retransmission at power $\alpha_{i}P_{i}$ and the remaining ${\bar{\alpha }}_{i}P_{i}$ is dedicated to the new arriving packet. After retransmission user 1 always decoded under the interference of the other user. Therefore its SINR during retransmission is given as ${\tilde{\gamma }}_{1}^{\left(1\right)}=\frac{\alpha_{1}P_{1}}{P_{2}+{\bar{\alpha }}_{1}P_{1}+1}$. The retransmission SINR of user 2 depends on the state of user 1. When receiver has two copies of user 2 and only a single copy of user 1 new transmission, then user 2 can be decoded first considering user 1’s new arriving packet as interference. In this situation, the power of interference signal of user 1 could be $P_{i}$ or ${\bar{\alpha }}_{i}P_{i}$ based on the state of user 1, i.e., $J_{1}=0$ or $J_{1}=1$, respectively. Consequently, the SINR of user 2 during retransmission, when $J_{1}=0$ and $J_{1}=1$, is ${\tilde{\gamma }}_{1}^{\left(2\right)}=\frac{\alpha_{2}P_{2}}{P_{1}+{\bar{\alpha }}_{2}P_{2}+1}$ and ${\tilde{\gamma }}_{2}^{\left(2\right)}=\frac{\alpha_{2}P_{2}}{{\bar{\alpha }}_{1}P_{1}+{\bar{\alpha }}_{2}P_{2}+1}$, respectively.

When the system is at state $J_{1}$, the SINR of user 1 is $\gamma_{1}^{\left(1\right)}=\frac{P_{1}}{P_{2}+1}$ as user 1 is decoded first. After removing interference of user 1, user 2 is decoded with SNR $\gamma_{1}^{\left(2\right)}=P_{2}$. When the system is at state $J_{2}$, $\gamma_{2}^{\left(1\right)}=\frac{P_{1}}{{\bar{\alpha }}_{2}P_{2}+1}$, which indicates that the previous packet of user 2 was recovered after retransmission and the new packet is the only interference. After removing packet of user 1, the new packet of user 2 experiences SNR $\gamma_{2}^{2}={\bar{\alpha }}_{2}P_{2}$. When the system is at state $J_{3}$, the previous packet of user 2 is not decoded so user 1 experiences a higher interference as $\gamma_{3}^{\left(1\right)}=\frac{P_{1}}{P_{2}+1}$. After removing the interference due to user 1 , user 2 only experiences interference from its retransmission packet, i.e., $\gamma_{3}^{\left(2\right)}=\frac{{\bar{\alpha }}_{2}P_{2}}{\alpha_{2}P_{2}+1}$. When the system is at state $J_{4}$, $\gamma_{4}^{\left(1\right)}=\frac{{\bar{\alpha }}_{1}P_{1}}{{\bar{\alpha }}_{2}P_{2}+1}$, $\gamma_{4}^{\left(2\right)}=\gamma_{2}^{2}$, since for both users previous packets are decoded successfully, and their interference is removed. At state $J_{5}$, user 2 is in error; therefore user 1 is decoded under its interference as $\gamma_{5}^{\left(1\right)}=\frac{{\bar{\alpha }}_{1}P_{1}}{P_{2}+1}$, $\gamma_{5}^{\left(2\right)}=\frac{{\bar{\alpha }}_{2}P_{2}}{\alpha_{2}P_{2}+1}$. Finally, when the system is at state $J_{6}$ user 1 is also not decoded so it causes interference for user 2, i.e., $\gamma_{6}^{\left(1\right)}=\frac{{\bar{\alpha }}_{1}P_{1}}{P_{2}+\alpha_{1}P_{1}+1}$ $\gamma_{6}^{\left(2\right)}=\frac{{\bar{\alpha }}_{2}P_{2}}{\alpha_{1}P_{1}+\alpha_{2}P_{2}+1}$. □

**Remark** **1.** *Let $\Pi =[\pi_{u\to v}]$ denotes the state transition matrix for the D-NOMA-HARQ system for 2 users and 1 retransmission. $P_{\mathrm{stat}}={\left[p_{1},\cdots ,p_{6}\right]}^{T}$ denotes the stationary distribution corresponding to the MM in Figure 2. The PER of user i , denoted by $\xi_{i}$, is given by*

(5) $$\begin{array}{c} \xi_{i}=\sum_{u\in E_{i}}p_{u}, \end{array}$$

*where $E_{1}=\left\{6\right\}$ and $E_{2}=\left\{3,5,6\right\}$. This follows directly from the fact that the stationary distribution of the system can be characterized by the eigenvector of matrix $\Pi^{T}$, corresponds to eigenvalue 1 and the PER is simply the stationary probability of user i being in the error state.*

**Remark** **2.** *With D-NOMA-HARQ the throughput of user i , denoted by $\eta_{i}\left(n,k_{i}\right)$, is accordingly given by*

(6) $$\begin{array}{c} \eta_{i}\left(n,k_{i}\right)=\frac{k_{i}\left(1-\xi_{i}\right)}{n}. \end{array}$$

*This is because with D-NOMA-HARQ, user i sends a new packet of length n with $k_{i}$ message bits in each time slot and received correctly at the receiver with the error rate $\xi_{i}$.*

### 3.2. Packet Delivery Delay Profile of D-NOMA-HARQ

As with D-NOMA-HARQ there is no queuing, each user sends at most N+1 packets when *N* packets are scheduled for the transmission. The delay profile of each user can be characterized as follow

(7) $$\begin{array}{c} D_{i}^{D}\left[d\right]=\sum_{u\in S_{i}}p_{u}\delta \left[d-N\right]+\left(1-\sum_{u\in S_{i}}p_{u}\right)\delta \left[d-N-1\right], \end{array}$$

where $S_{1}=\left\{1,2,3\right\}$ and $S_{2}=\left\{1\right\}$ denote the states of user 1 and 2, respectively, when a packet is successful without any retransmission.

### 3.3. Packet Delivery Delay Profile of S-NOMA-HARQ

Authors in [22] evaluated S-NOMA-HARQ with single retransmission and derived the PER ([22], Equation (14)) and throughput ([22], Equation (17)) for a given power allocation ratio. In particular, packet success probability of user *i* without retransmission denoted by $p_{s_{i}}$ is derived in ([22], Equation (16)). Since each retransmission delays the transmission of the new packet by one time slot, the delay profile of S-NOMA-HARQ for delivering *N* packets can be calculated as follows

(8) DiS[d]=∑j=0NNjpsij(1−psi)N−jδ[d−2N+j].

Because each packet of user *i* will be successful with single transmission with probability $p_{s_{i}}$, and each retransmission causes delay of a time slot with probability $1-p_{s_{i}}$. In S-NOMA-HARQ, since packets are orthogonal to each other, the packet delivery delay follows a binomial distribution.

### 3.4. Generalized $N_{u}$ User Setup

We can extend the model for general $N_{u}$ users. When single retransmission is allowed, each *i*-th user, $1\leq i\leq N_{u}$, can have $J_{i}\in \left\{0,1,e\right\}$, packet states. Consequently, there will be maximum $3^{N_{u}}$ vector states denoted as $J_{u}=\left[J_{1},J_{2},\cdots ,J_{N_{u}}\right]$, $u=[1,\cdots ,3^{N_{u}}]$. We use SIC decoding, where users are decoded based on their level of received powers. We assume user *i* is relatively closer to the BS than user *j*, when j>i. For example user 1 is considered near user, and user 2 is considered far user. We assume that with an equal number of received packets, the total received power of user *i* is always higher than user *j*. Therefore, the transmit power constraint of each user is implemented as $w_{j}\leq w_{i}$ and $\alpha_{i}\leq \alpha_{j}$. $w_{i}$ is the fraction of total received power from user *i*, i.e., $\sum_{i=1}^{N_{u}}w_{i}=1$ and $\alpha_{i}$ is the power of retransmitting signal, i.e., $\alpha_{i}\in \left[0,1\right]$. Therefore, when the *i*-th user is in error, i.e., $J_{i}=e$ the *j*-th user also cannot be decoded, i.e., $J_{j}=e$, where $i<j\leq N_{u}$. As a result, if the BS failed to decode user 1, then all the subsequent users would also fail the decoding due to their less received power than user 1. Similarly, if a user is successfully decoded only after retransmission, no subsequent user can succeed with single transmission. This assumption reduces the total number of states in the Markov model. The reduction in the maximum number of Markov model states denote as *U* is significant, for example, from 9 to 6 and 27 to 10 for $N_{u}=2$ and $N_{u}=3$, respectively.

Lemma 1 can be extended to the general $N_{u}$ users case. Lets consider a Markov model whose states are denoted as $J_{u}$, for u=[1,⋯,U], where *U* is the total number of states. Let $P_{\ell }\left(i,u\right)$ denotes the probability of user *i* transits to state *ℓ*, i.e., $J_{i}=\ell$, where ℓ∈{0,1,e}, when the system state is $J_{u}$. Note that each $J_{u}$ state corresponds to a specific packet state of user *i*, i.e., $J_{u}=\left[J_{i},\cdots ,J_{N_{u}}\right]$. For example when $N_{u}=2$, $J_{1}=\left[0,0\right]$ and $J_{6}=\left[e,e\right]$. The state transition probability for states $J_{u}$, is the product of marginal probabilities of each *i*-th user’s state $J_{i}=\ell$. We can define the state transition probabilities of a general $N_{u}$ user setup. When a user *i*, ( $1\leq i\leq N_{u}$), is decoded with a single transmission, its probability is given as $P_{0}\left(i,u\right)=1-\epsilon \left(\gamma_{u}^{\left(i\right)}\right)$. Consequently, user *j*, that comes next in decoding order can be decoded with probabilities $P_{0}\left(j,u\right)=1-\epsilon \left(\gamma_{u}^{\left(j\right)}\right)$, $P_{1}\left(j,u\right)=\epsilon \left(\gamma_{u}^{\left(j\right)}\right))-\epsilon \left(\gamma_{u}^{\left(j\right)},{\tilde{\gamma }}_{z}^{\left(j\right)}\right)$ and $P_{e}\left(j,u\right)=\epsilon \left(\gamma_{u}^{\left(j\right)},{\tilde{\gamma }}_{z}^{\left(j\right)}\right)$, respectively, for being in state $J_{j}=0$, $J_{j}=1$ and $J_{j}=e$. If the *i*-th user is recovered after retransmission, i.e., $J_{i}=1$, whose probability is $P_{1}\left(i,u\right)=\epsilon \left(\gamma_{u}^{\left(i\right)}\right))-\epsilon \left(\gamma_{u}^{\left(i\right)},{\tilde{\gamma }}_{z}^{\left(i\right)}\right)$, the subsequent user *j* can only have two states, i.e., $J_{j}=1$ and $J_{j}=e$, with probabilities $P_{1}\left(j,u\right)=1-\epsilon \left(\gamma_{u}^{\left(j\right)},{\tilde{\gamma }}_{z}^{\left(j\right)}\right)$ and $P_{e}\left(j,u\right)=\epsilon \left(\gamma_{u}^{\left(j\right)},{\tilde{\gamma }}_{z}^{\left(j\right)}\right)$, respectively. Finally, when decoding of user *i* is failed, i.e., $J_{i}=e$, which happens with probability $P_{e}\left(i,u\right)=\epsilon \left(\gamma_{u}^{\left(i\right)},{\tilde{\gamma }}_{z}^{\left(i\right)}\right)$, subsequent users would definitely fail the decoding, i.e., $P_{e}\left(j,u\right)=1$. At $N_{u}=3$ when all the users are successfully decoded with single transmission, the system transits to stat $J_{v}=\left[0,0,0\right]$. In general, the system transits from state $J_{u}$ to $J_{v}$ with probabilities as follows:

(9) $$\begin{array}{c} \pi_{u\to v}=\prod_{i=1}^{N_{u}}P_{J_{i}}\left(i,u\right),\;\left[J_{1},\cdots ,J_{N_{u}}\right]=J_{v}, \end{array}$$

where $\gamma_{u}^{\left(i\right)}$ and ${\tilde{\gamma }}_{z}^{\left(i\right)}$ are the SINR during first transmission and retransmission.

Now, we provide general guidelines to calculate the associated SINRs of the state transition probabilities for general $N_{u}$ users. The SINRs can be calculated based on the SIC decoding order. In general, a user with the highest received power is decoded first under the interference of other users with less power. Furthermore, if a user’s message is decoded successfully, its interference is removed; otherwise, it causes interference. Furthermore, when BS receives more copies due to the retransmission of a user, it is given priority over usual decoding order. This is because, with more copies, a weaker user could be decoded with better quality than a strong user. For example, when user *j* is retransmitting and has one more packet due to single retransmission than user *i*, it is decoded prior to user *i*. Consequently, if user *j* is decoded successfully its interference is eliminated, and user *i* experience less interference. These different states of user refer to the typical state in the Markov model dented as $J_{u}$. In general, SINR of user *i* during its first transmission is given as:

(10) $$\begin{array}{c} \gamma_{u}^{\left(i\right)}=\frac{\bar{\alpha_{i}}P_{i}}{\alpha_{i}P_{i}+\sum_{j}P_{j}-\sum_{k}\alpha_{k}P_{k}+1},\;\forall j\in I_{j},k\in I_{k} \end{array}$$

where $1\leq i\leq N_{u}$ and $I_{j}\in \left\{i+1,\cdots ,N_{u}+1\right\}$, $P_{N_{u}+1}=0$, $\alpha_{N_{u}+1}=0$ for notation consistency and $I_{k}\subseteq I_{j}$ is the set of indices referring to users that are successfully decoded after retransmission. Moreover, $P_{j}-\alpha_{j}P_{j}=\bar{\alpha_{j}}P_{j}$. The $\alpha_{i}P_{i}$ term in the denominator of (10) is the interference caused due to *i*-th user’s previously retransmitting packet. This would be zero if the packet has been decoded successfully. For example, when $N_{u}=2$$\gamma_{4}^{\left(1\right)}$ and $\gamma_{5}^{\left(1\right)}$ shows this case in Lemma 1. If there is no retransmitting packet, then SINR changes due to $\bar{\alpha_{i}}=1$, as shown in $\gamma_{2}^{\left(1\right)}$ in Lemma 1 for $N_{u}=2$ case. Moreover, $\sum_{k}\alpha_{k}P_{k}$ is the interference removed due to successfully decoding users with index set $I_{k}$. However, if the decoding failed then the interference cannot be removed. Consequently, the SINR could change to $\gamma_{u}^{\left(i\right)}=\frac{P_{i}}{\sum_{j}P_{j}+1}$$\forall j\in I_{j}$, which shows that the BS decodes the message of the *i*-th user under the interference of subsequent *j* users with maximum power.

User *i* always conducts its retransmission under the interference of its own new arriving packet and packets of subsequent users. The general expression of SINR of user *i* during retransmission can be written as:

(11) $$\begin{array}{c} {\tilde{\gamma }}_{z}^{\left(i\right)}=\frac{\alpha_{i}P_{i}}{\bar{\alpha_{i}}P_{i}+\sum_{j=i+1}^{N_{u}}P_{j}+1},\;\;\;z\in \left\{0,1,2\right\}, \end{array}$$

where $1\leq i\leq N_{u}$. Upon successful decoding of user *i* after retransmission, BS can remove its interference amounts to $\alpha_{i}P_{i}$, ∀i<j for decoding user *j*. With all the state transition probabilities, the stationary distribution corresponding to the MM can be calculated using standard methods. Finally, accumulating the stationary probabilities corresponding to the erroneous packet states of each user $\left(J_{i}=e\right)$, the PER and throughput can be obtained for $N_{u}$ users similar to Equations (5) and (6).

**Remark** **3.** *Note that, in the general $N_{u}$ users case, we keep the maximum allowed retransmission of failing packet to one. In URLLC, the number of retransmissions is kept small to minimize the delay. However, the model in this paper can be extended for maximum M retransmission case with ${\left(M+2\right)}^{N_{u}}$ MM states, where in general, the success probability of the i-th user at the m-th retransmission can be written as $\epsilon \left(\left[\gamma_{1}^{\left(i\right)},\ldots ,\gamma_{m-1}^{\left(i\right)}\right]\right)-\epsilon \left(\left[\gamma_{1}^{\left(i\right)},\ldots ,\gamma_{m}^{\left(i\right)}\right]\right)$. Then, various combinations of the product of m terms according to individual user’s states defines the state transition matrix. We skip the details to keep the presentation simple.*

## 4. Numerical Results

In the simulations, we consider an uplink NOMA with two users and allow a maximum of 1 retransmission using HARQ. Using the MM, the PER, delay, and throughput performance are analyzed for each user with various transmission rates $R_{i}$, SNRs, power-splitting ratios $w_{i}$ for NOMA, and $\alpha_{i}$ for non-orthogonal HARQ and length of packet *n*. For simplicity of presentation, we set $k_{1}=k_{2}=k$, unless specified otherwise. For simulation, we primarily focused on the CC-HARQ scheme for the detailed analysis and compared it with IR-HARQ for some special cases. When IR-HARQ is employed, the retransmission parity length can be adjusted at the cost of a slight signaling overhead. However, this can increase packet-level latency. In contrast, when CC-HARQ is employed, the whole packet is repeated to increase reliability. Therefore, CC-HARQ is more suitable for URLLC applications due to a simpler design with less signaling overhead.

### 4.1. Effect of SNR and Rate on PER, Throughput, and Delay

**Figure 3:**
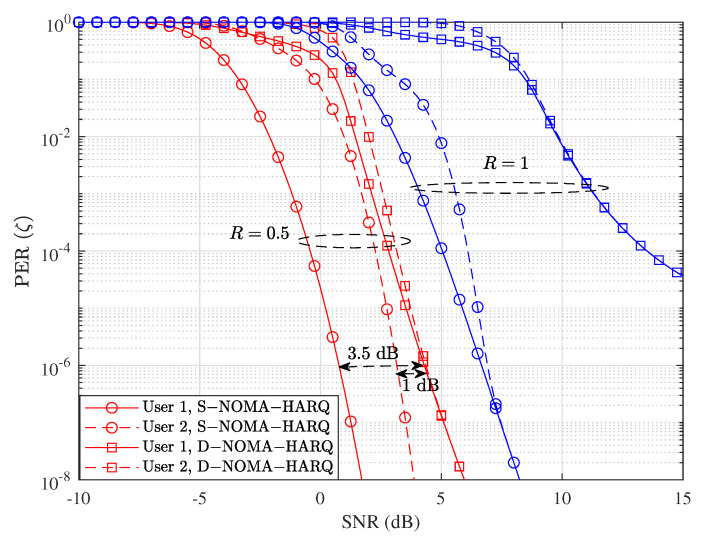
Packet error rate performance comparison between D-NOMA-HARQ and S-NOMA-HARQ with $w_{1}=0.6$ ($w_{2}=1-w_{1}$) with CC-HARQ, when m=1, $\alpha_{1}=0.5$, and $\alpha_{2}=0.4$, at various rates.

Figure 3 shows error rate performance comparison of proposed D-NOMA-HARQ and S-NOMA-HARQ with different SNRs and rates. We fix the packet length n=100 and vary k=100 and k=50 to model rate R=1 and R=0.5, respectively. As shown in this figure, the PER performance of D-NOMA-HARQ is worse than S-NOMA-HARQ, and there exists an SNR performance gap. More specifically, the SNR gaps for target PER of $10^{-4}$ at rate R=1 are about 6 dB and 8 dB for user 1 and 2, respectively. However, by reducing the rate to R=0.5, the PER performance gap reduces to about 3 dB and 1 dB for user 1 and 2, respectively, for the target PER performance range $10^{-4}$ to $10^{-6}$. This is because D-NOMA-HARQ is designed for target delay performance and uses less power and time slots by serving retransmission requests non-orthogonally with new arriving packets, whereas in S-NOMA-HARQ the retransmission is conducted with full power in the new time slot. Thus overall, D-NOMA-HARQ conducts its transmissions with efficient power and bandwidth utilization. Another cause of PER performance loss is that in D-NOMA-HARQ, non-orthogonal sharing of packets causes interference if SIC is unsuccessful. On the other hand, S-NOMA-HARQ allocates full resources, i.e., time slots, and the SNR is same for its transmissions and retransmissions. Fortunately, with a lower rate, i.e., R=0.5, the non-orthogonal retransmission and new transmission can be decoded with a higher success rate using SIC; therefore, the SNR gap between D-NOMA-HARQ and S-NOMA-HARQ decreases.

**Figure 4:**
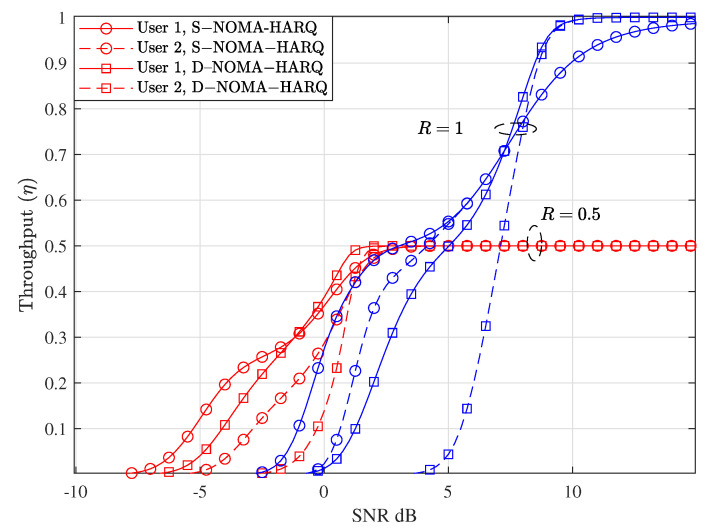
Throughput performance comparison between D-NOMA-HARQ and S-NOMA-HARQ with $w_{1}=0.6$ and $w_{1}=0.6$ ($w_{2}=1-w_{1}$) with CC-HARQ, when m=1, $\alpha_{1}=0.5$, and $\alpha_{2}=0.4$, at various rates.

Figure 4 shows the throughput versus SNR performance comparison between S-NOMA-HARQ and the proposed D-NOMA-HARQ at two different rates, i.e., R=1 and R=0.5. As can be seen in the figure, S-NOMA-HARQ achieves higher throughput than D-NOMA-HARQ, when the SNR is low. With D-NOMA-HARQ at low SNRs, SIC decoding of packets is more erroneous because of overlapping transmission and retransmission packets. Whereas, the reliability of S-NOMA-HARQ is superior to D-NOMA-HARQ because HARQ retransmissions are conducted independently to regular transmission with maximum power and separate time slot. However, as the SNR increases and the packet can be recovered using SIC more reliably, excessive retransmission of S-NOMA-HARQ results in throughput saturation. On the other hand, D-NOMA-HARQ maintains a steady gain in throughput with SNR and eventually attains the same level of throughput as achieved with S-NOMA-HARQ. Note that D-NOMA-HARQ throughput performance is better than S-NOMA-HARQ at a specific rate point and SNR region, such as R=1 and SNRs from 8 dB to 15 dB. This throughput gain of D-NOMA-HARQ over S-NOMA-HARQ can be seen in Figure 4 clearly, at SNR = 10 dB and R=1. Because at this SNR and rate, the maximum number of retransmitted packets can be recovered using SIC with very high reliability. Finally, the throughput performance of D-NOMA-HARQ and S-NOMA-HARQ eventually becomes similar, when the SNR is very high, such that no retransmission is required and all the packets are successful with only a single transmission.

**Figure 5:**
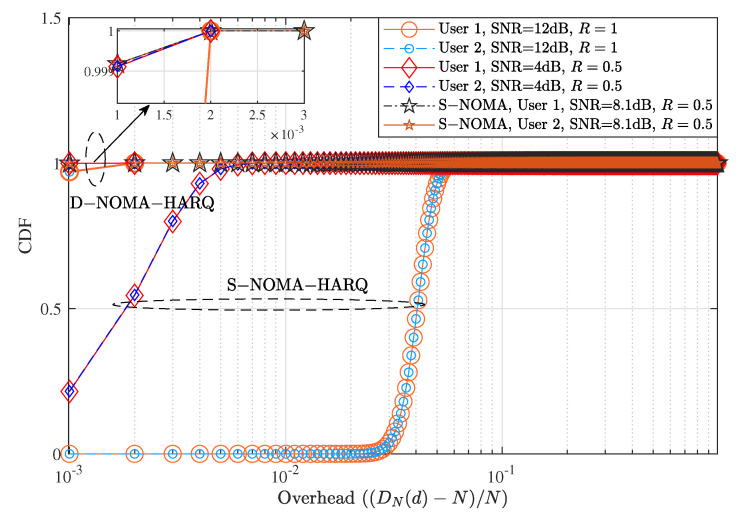
Delay performance comparison between D-NOMA-HARQ and S-NOMA-HARQ with $w_{1}=0.6$ ($w_{2}=1-w_{1}$) with CC-HARQ when m=1, $\alpha_{1}=0.5$, and $\alpha_{2}=0.4$, at various SNRs and rates.

We present the delay performance comparison between proposed D-NOMA-HARQ and S-NOMA-HARQ in Figure 5. We assume that N=1000 packets are scheduled to be transmitted, and each packet has a specific deadline to reach the receiver. We assume feedback, decoding, and other processing delays to be zero and only account for the retransmission delay. The overall delay of 1000 packets is normalized to 1. Therefore if all the packets are delivered by their respective deadline, the delay overhead is zero. However, if a packet is received after one unit of delay due to retransmission, it incurs a delay overhead. With a specific retransmission rate *R* and SNR, each scheduled user has an average PER reliability, as can be seen in Figure 3. On the contrary, packet-level delay derived in (8) is not an average delay measure and depends upon the number of scheduled packets. However, the error rate of each of *N* scheduled packets can be found by (5). The D-NOMA-HARQ design does not allow a packet to be delayed by more than one time slot. In contrast, each retransmission in S-NOMA-HARQ delays all the subsequent packets by one slot. Therefore the delay performance of D-NOMA-HARQ is much better than S-NOMA-HARQ. In both S-NOMA-HARQ and D-NOMA-HARQ a packet is discarded if the maximum retransmission limit is reached and the receiver is unable to decode.

As shown in Figure 3, at rate R=1 and SNR = 12 dB, S-NOMA-HARQ achieves PER $\approx 10^{-8}$; however, it delays a significantly high number of packets (shown in Figure 5). In contrast, D-NOMA HARQ provides reliability of PER $\approx 10^{-4}$ with a much lower packet-level latency guarantee. Also, by increasing the SNR or reducing the rate, D-NOMA-HARQ can provide the desired packet-level performance without violating the packet level delay deadlines. For example, as can be seen in Figure 3, at rate R=0.5 with SNR ≈ 4 dB, D-NOMA-HARQ achieves PER $\approx 10^{-6}$. In contrast, S-NOMA-HARQ achieves a much better PER. However, Figure 5 shows that at rate R=0.5 and SNR = 4 dB, the delay performance of S-NOMA-HARQ is much inferior than D-NOMA-HARQ. More specifically, S-NOMA-HARQ achieves the delay performance of D-NOMA-HARQ at R=0.5 at SNR = 8.1 dB (additional 4.1 dB SNR for each user) as shown in Figure 5. Whereas D-NOMA-HARQ achieves the PER performance of S-NOMA-HARQ at R=0.5 with only 1 dB and 3.5 dB additional SNR for user 1 and 2, respectively, as shown in Figure 3. This proves that D-NOMA-HARQ is much superior to S-NOMA-HARQ in providing a certain level of PER reliability and packet-level delay performance.

### 4.2. Effect of Packet Length *n* on PER and Throughput

**Figure 6:**
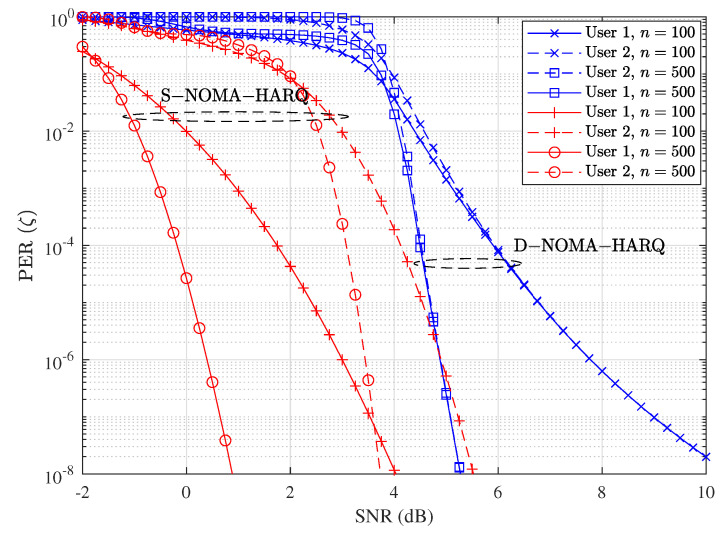
Packet error rate performance comparison between D-NOMA-HARQ and S-NOMA-HARQ at setting $w_{1}=0.6$, ($w_{2}=1-w_{1}$) with CC-HARQ when m=1, $\alpha_{1}=0.5$, $\alpha_{2}=0.4$, for various packet lengths *n*.

One can observe the effect of increasing the packet length while keeping the rate R=k/n fixed. Figure 6 shows the PER performance variation of the proposed D-NOMA-HARQ and S-NOMA-HARQ with the increasing packet length. We use two different packet lengths as n=100 and n=500. It is clear from the figure that the reliability performance of both schemes increases with the packet length. This performance gain with increasing length follows the normal approximation and finite packet length assumption [10]. When the packet length is short, i.e., n=100, the reliability gain with SNR is small due to poor SIC decoding capability. When the packet length increases, the receiver can utilize longer codewords to decode the packets better and remove the successfully decoded packet from non-orthogonal packets using SIC. Therefore, when the packet length is large, i.e., n=500, the PER performance of D-NOMA-HARQ steadily improves with SNR.

**Figure 7:**
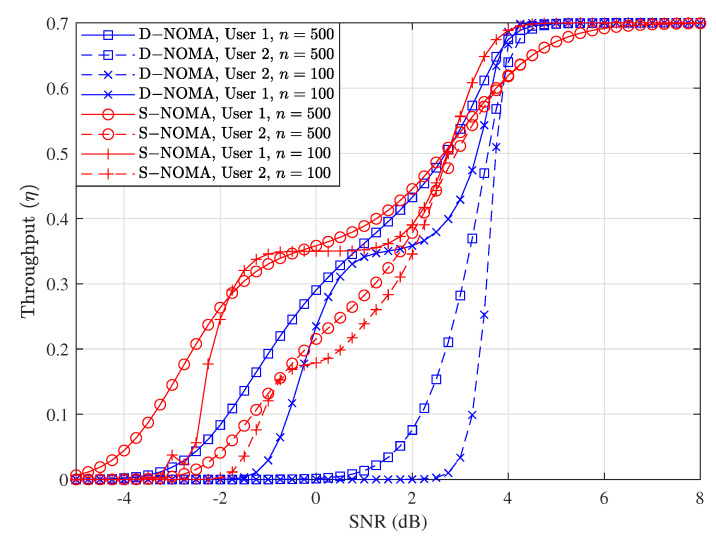
Throughput performance comparison between D-NOMA-HARQ and S-NOMA-HARQ at setting $w_{1}=0.6$, ($w_{2}=1-w_{1}$) with CC-HARQ when m=1, $\alpha_{1}=0.5$, $\alpha_{2}=0.4$, for various packet lengths *n*.

Figure 7 shows throughput performance of the proposed D-NOMA-HARQ and baseline S-NOMA-HARQ with packet lengths n=100 and n=500. As shown in this figure, the throughput performance of both schemes improves with increasing packet length, especially in the low SNR regime. When packet length is large, S-NOMA-HARQ performance starts to saturate earlier in the medium SNR range. This saturating performance is because, with S-NOMA-HARQ, the excessive retransmission penalty is larger when *n* is large. Since D-NOMA-HARQ conducts its retransmission by shared power and time resource, it does not incur retransmission overhead. Therefore, D-NOMA-HARQ achieves steady throughput performance gain even when the packet length is large. Nevertheless, we see user 1’s saturating throughput performance trend with D-NOMA-HARQ. This is mainly because of excessive retransmission power fraction value for user 1, i.e., $\alpha_{1}$, when *n* is larger. In practice, the user needs to adopt the power of retransmission with packet length. Therefore, when the packet length is larger, smaller values of retransmission powers $\alpha_{i}$ are sufficient and vice versa. The effect of power-sharing parameters and their optimization are discussed in detail in the following

### 4.3. PER and Throughput Performance for different $R_{1}$ and $R_{2}$

In practical systems, users adopt the transmission rate according to their respective channel quality to increase reliability. Each user can transmit at a different rate according to their respective quality of service by changing *k*. We set $R_{1}=k_{1}/n$ and $R_{2}=k_{2}/n$ by fixing the blocklength *n* and varying $k_{1}$ and $k_{2}$ for user 1 and user 2, respectively. We assume $k_{1}<k_{2}$ to allow user 1 with higher decoding reliability than user 2.

**Figure 8:**
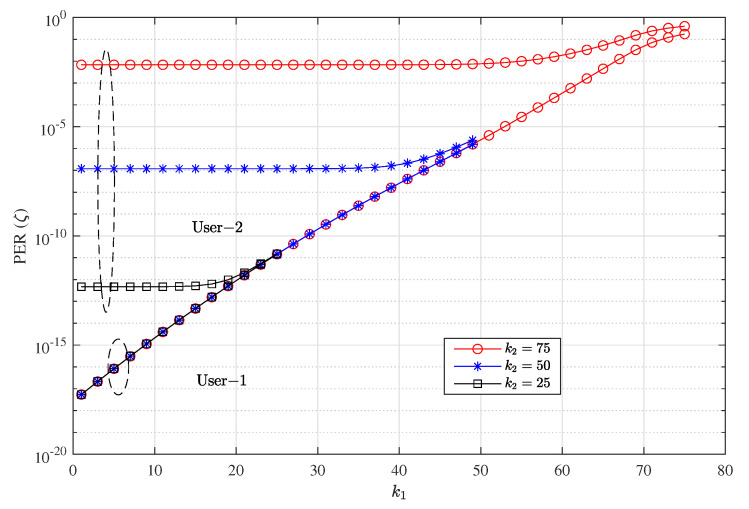
Packet error rate performance of each user with different *k* for each user at setting $w_{1}=0.6$, $w_{2}=0.4$ with CC-HARQ when m=1, $\alpha_{1}=0.5$, $\alpha_{2}=0.4$, for packet lengths n=100 and SNR = 4 dB.

**Figure 9:**
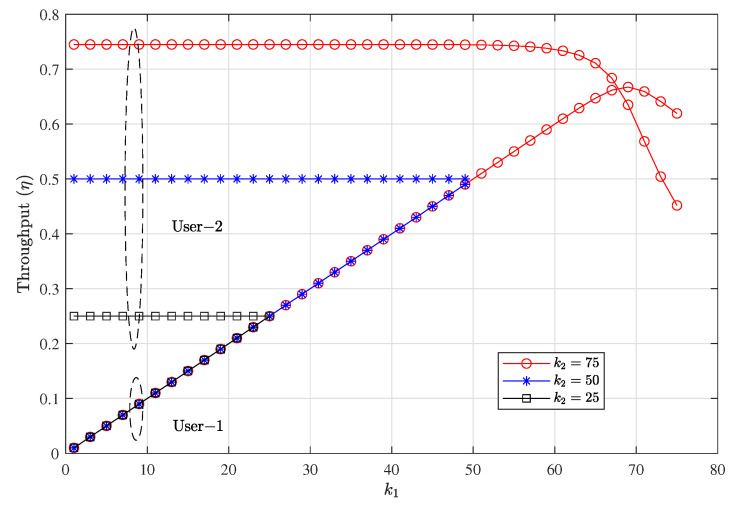
Throughput variation with different *k* for each user at setting $w_{1}=0.6$, ($w_{2}=1-w_{1}$) with CC-HARQ when m=1, $\alpha_{1}=0.5$, $\alpha_{2}=0.4$, for packet lengths n=100 and SNR = 4 dB.

Figure 8 and Figure 9 show the PER and throughput performance of each user with various $k_{1}$ and $k_{2}$. As can be seen in the figure, a higher value of $k_{1}$ leads to lower PER reliability. The figures show that by choosing different *k* for each user, different levels of reliability can be achieved. The PER is reduced with smaller values of *k*; however, it reduces the maximum achievable throughput as well as can be seen in Figure 9 . This is because at SNR = 4 dB and n=100 a higher rate can be chosen with an acceptable PER reliability. Also, the throughput steadily increases with $k_{1}$; however, setting $k_{1}$ very high decreases the PER reliability and throughput performance starts to saturate. We can see in Figure 8 that at $k_{j}\geq 75$ results in severe PER degradation, and consequently, throughput performance starts to saturate for both users. Since user 1 is operating at higher transmission power than user 2 the throughput loss is higher in user 2 when $k_{j}$ is very high.

### 4.4. PER. Throughput, and Delay Performance When $N_{u}=3$

**Figure 10:**
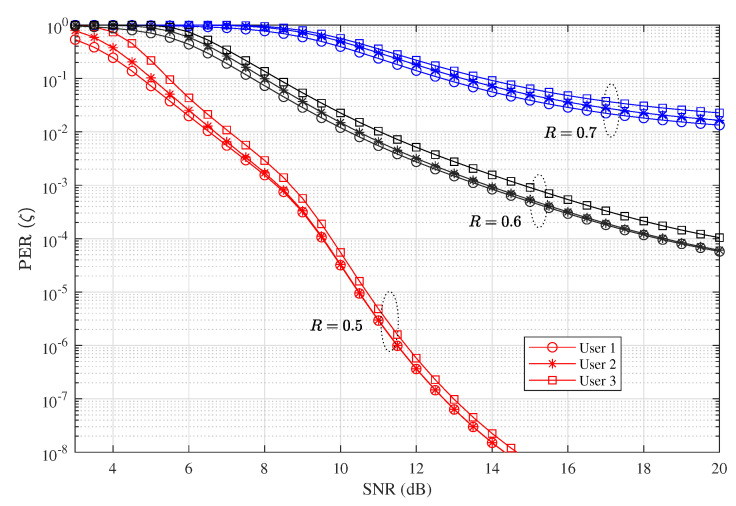
PER performance of D-NOMA-HARQ with CC-HARQ serving maximum 3 users at $w_{1}=0.43$, $w_{2}=0.37$, $w_{3}=0.2$, $\alpha_{1}=0.8$, $\alpha_{2}=0.6$, $\alpha_{3}=0.4$, and n=200.

**Figure 11:**
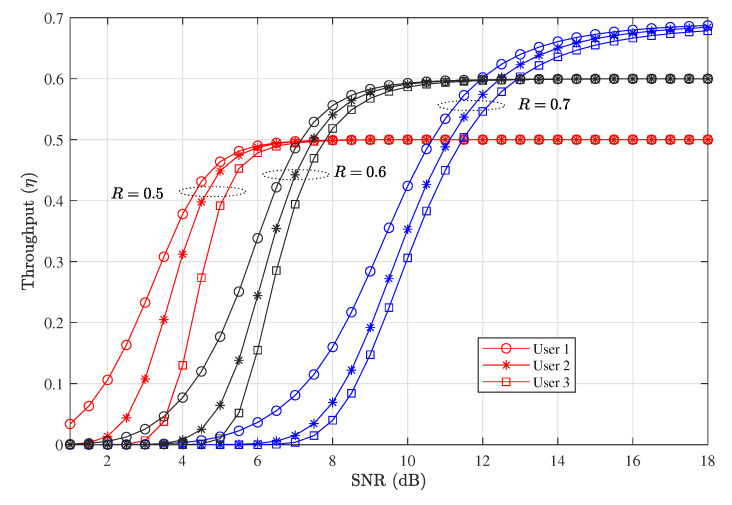
Throughput performance of D-NOMA-HARQ with CC-HARQ serving maximum 3 users at $w_{1}=0.43$, $w_{2}=0.37$, $w_{3}=0.2$, $\alpha_{1}=0.8$, $\alpha_{2}=0.6$, $\alpha_{3}=0.4$, and n=200.

Figure 10 and Figure 11 show respectively the PER and throughput performance of D-NOMA-HARQ when $N_{u}=3$. We show the performance variation with different code rates *R*. As can be seen in Figure 10, when the code rate is high, the PER performance is poor in low SNRs and reduces slowly in the medium and high SNRs. The corresponding throughput is also poor in low SNRs and increase gradually when R=0.7. This is because when the number of users are increased, each user experiences relatively higher interference. However, by reducing the rate, i.e., R=0.6 and R=0.5, the early saturation of PER can be avoided. This results in higher throughput in low SNRs. Therefore, with increasing the number of users with D-NOMA-HARQ, proper code rates should be chosen to achieve the target PER and throughput for all users.

### 4.5. Effect of Power-Sharing Parameters, $w_{i},\alpha_{i}$, on PER, Throughput, and Delay

**Figure 12:**
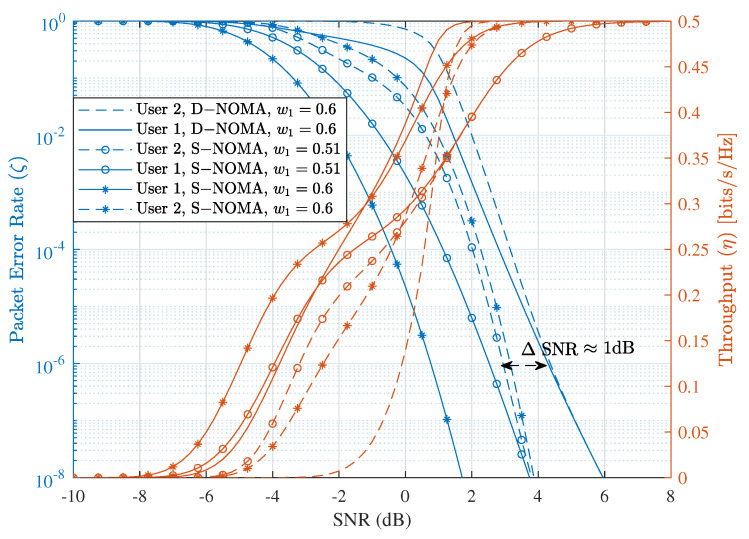
PER and throughput performance comparison between D-NOMA-HARQ setting $w_{1}=0.6$, and S-NOMA-HARQ setting $w_{1}=0.51$, 0.6 ($w_{2}=1-w_{1}$) with CC-HARQ when m=1, $\alpha_{1}=0.5$, $\alpha_{2}=0.4$, n=100, k=50.

We use $w_{1}=0.6$ and 0.51 ($w_{2}=1-w_{1}$) to show performance variation of each user with different power fractions. The parameter $w_{i}$ indicates the power fraction of NOMA paired users sending packets on the same channel with either S-NOMA-HARQ or D-NOMA-HARQ. In addition to $w_{i}$, $\alpha_{i}$ is used to indicate the power fraction of the retransmission packet for D-NOMA-HARQ. First, we present the effect of parameter $w_{i}$ on the performance of S-NOMA-HARQ in the following. Figure 12 shows the PER of S-NOMA-HARQ and D-NOMA-HARQ with different $w_{i}$. As shown in this figure, with S-NOMA-HARQ, when the power difference between users is small, i.e., $w_{1}=0.51$, both users achieve similar PER performance as the SNR increases. More specifically, at SNR ≈ 3 dB, both users achieve PER of about $10^{-6}$ with S-NOMA-HARQ. However, by increasing the power difference between users ($w_{1}=0.6$), user 1 achieves much higher reliability at the cost of a small increase of PER of user 2. When the power difference between users is more considerable ($w_{1}=0.6$), the SIC is better, resulting in PER and throughput performance improvement. However, increasing $w_{i}$ too much leads to PER performance disparity among users, where a user with higher power get much higher reliability, while the PER reliability of other user decreases. More specifically, at $w_{1}=0.6$, the PER of user 1 is less than $10^{-8}$ while user 2’s PER is slightly higher than $10^{-6}$. On the other hand, with D-NOMA-HARQ, a larger $w_{i}$ can be placed between users to improve the SIC decoding, while user performance disparity can be reduced with the adjustment of parameter α. As shown in Figure 12, with D-NOMA-HARQ at $w_{1}=0.6$ both users achieve similar PER performance. Note that another drawback of S-NOMA-HARQ is that a slight increase in PER of a user results in much lower latency performance. Therefore, increasing $w_{i}$ too much is also prohibitive.

**Figure 13:**
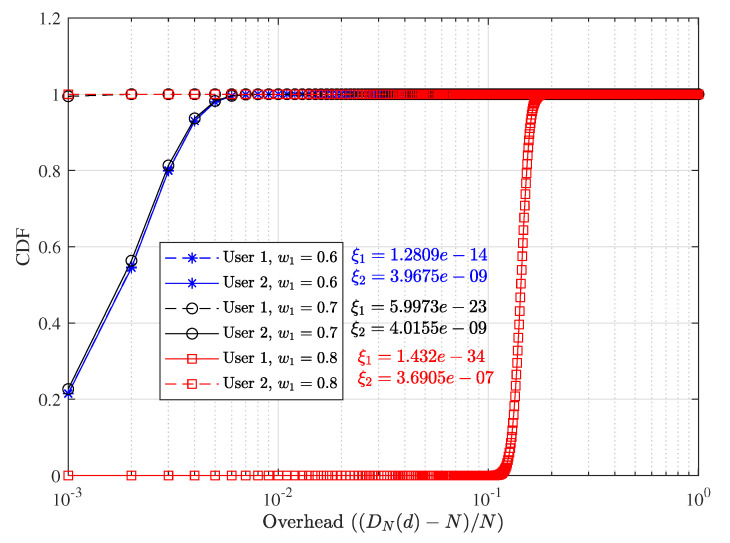
Effect of $w_{i}$ over delay performance of S-NOMA-HARQ with CC-HARQ at R=0.5 and SNR = 4 dB.

Figure 13 shows the delay performance of each user with S-NOMA-HARQ at SNR 4dB with their PER performance. Using higher power difference between users, i.e., larger $w_{1}$, the PER reliability of 1 user increases beyond the need while the other user suffers from slight PER performance degradation. However, the slight PER performance degradation with S-NOMA-HARQ results in significant packet-level latency performance degradation.

**Figure 14:**
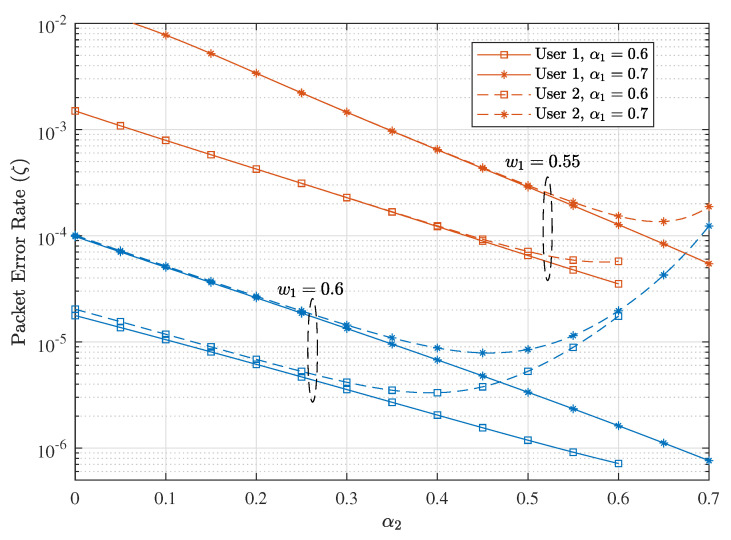
Effect of $w_{i}$, $\alpha_{1}$ and $\alpha_{2}$ on the PER performance of D-NOMA-HARQ with CC-HARQ at R=0.5 and SNR = 4 dB.

Next, we present the effect of parameters $w_{i}$ and $\alpha_{1}$ on the PER of D-NOMA-HARQ, when $N_{u}=2$. Figure 14 shows the PER performance of both users with various $w_{i}$ and $\alpha_{i}$ settings. Note that the choice of $w_{i}$ and $\alpha_{i}$ greatly affect the PER performance of D-NOMA-HARQ. By fixing $\alpha_{1}$ and $w_{1}$, $\alpha_{2}$ is varied to see the performance variation at different settings. As can be seen, at smaller values of $w_{1}$, e.g., 0.55, both users achieve similar PER performance; however, due to the small power difference among users at $w_{1}=0.55$, SIC is not performing well. By Increasing $w_{1}$ to 0.6, PER can be reduced further. Furthermore, when $\alpha_{i}$ is large, e.g., 0.7, excessive power is assigned to conduct retransmission, leaving insufficient power for new arriving packets to be decoded successfully. However, by reducing $\alpha_{i}$ to 0.6, a further PER reduction is achieved.

### 4.6. Optimization of $w_{i}$ and $\alpha_{i}$

We now consider an optimization problem to find the optimal power-splitting ratios, $w_{i}$ ($w_{2}=1-w_{1}$) and $\alpha_{i}$, to minimize the worst PER among both users when using D-NOMA-HARQ. For a given SNR and rate $R=k_{j}/n$, the optimization problem is summarized below:

(12) $$\begin{array}{cc} \min_{\left\{w_{1},\alpha_{1},\alpha_{2}\right\}} & \max_{i}\;\xi_{i}\\ s.t.\;\; & C_{1}.\;0\leq \alpha_{2}\leq \alpha_{1}\leq 1,\;\\ \; & C_{2}.\;0.5<w_{1}\leq 1, \end{array}$$

condition $C_{1}$ is to limit the search space for practical optimization, and $C_{2}$ is to allocate a higher power to user 1.

We numerically solved (12) by fixing $w_{i}$ and finding $\alpha_{1}$ and $\alpha_{2}$. Table 1 shows optimal values of $\alpha_{1}$ and $\alpha_{2}$ when R=0.5 at different SNRs and $w_{i}$. As can be seen in the Table, IR-HARQ performance is slightly better than CC-HARQ. Furthermore, as SNR increases, higher values of $\alpha_{i}$ can be chosen to improve the performance. Also, with a higher value of $w_{1}$, PER performance improves due to better SIC, with proper choice of $\alpha_{i}$. The parameter $w_{i}$ refers to user pairing. As BS is assumed to know the CSI of each user, it can choose $w_{i}$ by pairing user with specific power difference to meet the target PER requirements of each user. As shown in Table 1, when $w_{1}=0.55$, the PER of $10^{-6}$ can be achieved for both users by choosing $\alpha_{1}=\alpha_{2}=0.6$. The advantage of D-NOMA-HARQ is that this level of reliability can be achieved with much less latency compared with S-NOMA-HARQ.

Compared to standard NOMA, D-NOMA-HARQ has a higher SIC decoding complexity, which also incurs some delay in the decoding. This is because the S-NOMA-HARQ receiver performs SIC to separate the signals of different users, whereas, in D-NOMA-HARQ, retransmissions and regular packets of a user are also separated using SIC. However, the additional decoding delay is much less than the delay caused due to packet retransmissions, feedback, processing, and queuing delays. Fortunately, the SIC complexity can be significantly reduced using efficient parallel decoding techniques. However, the exact analysis is beyond the scope of this work and would be more relevant when actual encoder and decoder such as low-density parity-check codes (LDPC) are being used. The exact complexity analysis can be performed by adding associating SIC cost to the retransmission states of the MM in Figure 2. The analysis can be extended to incorporate the decoding delay simply by adding delay penalty at packet retransmission state J=1 of the MM. Furthermore, D-NOMA-HARQ requires multi-bits feedback signaling due to more received signals in a time slot during retransmission than S-NOMA-HARQ.

## 5. Conclusions

In this paper, we proposed a multiuser uplink strategy for delay-sensitive applications. NOMA was used to allow simultaneous transmission of users’ packets and also to allow retransmission to share resources with new arriving packets to limit delay. In this way, the target reliability is achieved without causing queuing to any of the users. We analyzed the throughput, PER, and delay performance of the proposed scheme by using a MM. We also defined and solved an optimization problem to find power-sharing parameters to minimize the PER. Results show that the proposed scheme significantly outperforms the standard NOMA-HARQ scheme in terms of the packet delivery delay.

## Figures and Tables

**Table 1:** Optimal parameters for D-NOMA-HARQ.

		CC-HARQ		IR-HARQ	
$w_{1}$	SNR	($\alpha_{1}$, $\alpha_{2}$)	$\left(\min\;\max_{i}\xi_{i}\right)$	$(\alpha_{1}$, $\alpha_{2})$	$\left(\min\;\max_{i}\xi_{i}\right)$
	3 dB	0.5, 0.5	0.00117	0.5 , 0.5	0.00058
0.55	4 dB	0.6, 0.57	$5.67\times 10^{-5}$	0.6, 0.57	$1.58\times 10^{-5}$
	5 dB	0.6, 0.6	$1.197\times 10^{-6}$	0.6, 0.6	$4.4\times 10^{-7}$
	3 dB	0.5, 0.3	0.000165	0.5, 0.3	$6.68\times 10^{-5}$
0.6	4 dB	0.55, 0.4	$3.023\times 10^{-6}$	0.6, 0.4	$1.03\times 10^{-6}$
	5 dB	0.6, 0.5	$5.839\times 10^{-8}$	0.6, 0.4	$1.22\times 10^{-8}$
	3 dB	0.6, 0.36	0.00058	0.7, 0.4	0.0003
0.65	4 dB	0.6, 0.45	$4.8\times 10^{-6}$	0.7, 0.46	$1.98\times 10^{-6}$
	5 dB	0.6, 0.44	$1.313\times 10^{-8}$	0.7, 0.46	$5.75\times 10^{-9}$

## Data Availability

Not applicable.
